# Atrial flutter complicating acute respiratory distress syndrome (ARDS): A rare case report

**DOI:** 10.1097/MD.0000000000036216

**Published:** 2024-01-05

**Authors:** Chukwuka Elendu, Dependable C. Amaechi

**Affiliations:** a University of California, Santa Cruz, United States; b Igbinedion University Okada, Okada, Nigeria.

**Keywords:** acute respiratory distress syndrome (ARDS), atrial flutter, case report, complication, rare case

## Abstract

**Rationale:**

This case report addresses a unique instance of atrial flutter complicating acute respiratory distress syndrome (ARDS), contributing a novel addition to the medical literature. The co-occurrence of these conditions sheds light on a rare clinical scenario that requires careful consideration.

**Patient Concerns:**

The patient exhibited symptoms of pronounced dyspnea, tachypnea, and hypoxemia. Clinical assessment revealed irregular heart rhythms, notably atrial flutter, alongside characteristic signs of ARDS, including bilateral pulmonary infiltrates and reduced lung compliance.

**Diagnoses and Interventions:**

After a comprehensive evaluation, the patient was diagnosed with atrial flutter complicating ARDS. Therapeutic measures encompassed antiarrhythmic agents, mechanical ventilation, and targeted ARDS management protocols. The intricate interplay between cardiac and respiratory factors necessitated a multidisciplinary approach.

**Outcomes:**

Throughout treatment, the patient’s respiratory distress gradually improved. Control of the atrial flutter was achieved, and oxygenation levels were restored within acceptable limits. This successful outcome underscores the significance of a well-coordinated treatment strategy in addressing complex cases like this.

**Lessons:**

This case highlights the importance of recognizing and managing the intricate relationship between cardiac arrhythmias such as atrial flutter and respiratory complications like ARDS. The successful management of this patient underscores the value of multidisciplinary collaboration and tailored therapeutic interventions. Practitioners should remain vigilant for such rare complications and consider this case a reminder of the potential complexities that can arise in critical care scenarios.

## 1. Introduction

The co-occurrence of atrial flutter and acute respiratory distress syndrome (ARDS) presents a rare and intriguing scenario in medical complexities. While both conditions individually demand focused clinical attention, their convergence offers a unique challenge requiring a comprehensive approach. This case report contributes to the medical literature by shedding light on this distinctive interplay and its management.

Atrial flutter, characterized by rapid and organized atrial contractions, has been extensively studied due to its association with cardiac dysfunction.^[1]^ On the other hand, ARDS is a well-recognized entity characterized by severe hypoxemic respiratory failure and widespread lung inflammation.^[2]^ Yet, the occurrence of atrial flutter complicating ARDS needs to be more sparsely documented, resulting in limited guidance for clinicians encountering such cases.

The rarity of this coexistence underscores the need for increased awareness among healthcare practitioners. Our report builds upon the existing literature by presenting a detailed account of a patient with atrial flutter superimposed on ARDS. We aim to enhance medical understanding and contribute to improved management strategies by discussing the diagnostic and therapeutic challenges encountered.

## 2. Patient information

- Pseudonym: Mr. Smith
- Age: 54
- Gender: Male
- Ethnicity: Caucasian
- Occupation: Accountant

Main symptoms: Mr. Smith presented with the following chief complaints:

- Severe dyspnea
- Rapid breathing (tachypnea)
- Profound hypoxemia

### 2.1. Medical, family, and psychosocial history

- Medical history: Mr. Smith had a history of hypertension managed with antihypertensive medications. He was also diagnosed with obstructive sleep apnea and used continuous positive airway pressure therapy.
- Family history: His family history of cardiovascular diseases or respiratory disorders was unremarkable.
- Psychosocial history: No significant psychosocial factors were noted.

### 2.2. Co-morbidities and genetic information

- Co-morbidities: Mr. Smith had a history of hyperlipidemia, hypertension, and obstructive sleep apnea.
- Genetic Information: No relevant genetic information was available.

### 2.3. Relevant past interventions and outcomes

- Mr. Smith had undergone cardiac catheterization 5 years ago, revealing no significant coronary artery disease. He was prescribed antihypertensive medications and advised lifestyle modifications, resulting in well-controlled blood pressure.
- The continuous positive airway pressure therapy effectively managed his obstructive sleep apnea, improving his sleep quality and reducing daytime fatigue.

## 3. Clinical findings – physical examination

Upon physical examination, several relevant findings were noted:

### 3.1. Respiratory system

- Increased respiratory rate (tachypnea) with a respiratory rate of 28 breaths per minute.
- Use of accessory muscles during breathing.
- Bilateral inspiratory crackles are heard upon auscultation of the lung fields.
- Decreased breath sounds at the lung bases are indicative of reduced lung compliance.

### 3.2. Cardiovascular system

- Irregular heart rhythm with rapid atrial contractions, identified as atrial flutter.
- Heart rate of 110 beats per minute.
- Blood pressure within normal limits.

### 3.3. Skin and peripheral circulation

- Peripheral cyanosis was noted, particularly in the fingers and toes.
- No significant edema in the extremities.

### 3.4. Neurological system

- Alert and oriented to time, place, and person.
- No signs of neurological deficits.

## 4. Timeline depicting the essential milestones related to Mr. Smith diagnosis and interventions

**Table:** 

Day 1: Mr. Smith was hospitalized with severe dyspnea, tachypnea, and hypoxemia. Physical examination reveals irregular heart rhythms and bilateral inspiratory crackles. Initial diagnostic tests include an electrocardiogram and chest X-ray. Day 2: An echocardiogram confirms the diagnosis of atrial flutter. Arterial blood gas analysis indicates severe hypoxemia. Mechanical ventilation is initiated to support oxygenation. Day 4: Comprehensive workup confirms the diagnosis of atrial flutter complicating acute respiratory distress syndrome (ARDS). Multidisciplinary team discussion outlines the treatment plan. Day 7: Antiarrhythmic medication is initiated to control atrial flutter. Pronounced improvement in heart rate and rhythm is observed. Day 10: Despite improvements in cardiac rhythm, oxygenation remains a challenge. Positive end-expiratory pressure (PEEP) is adjusted to optimize lung compliance. Day 14: Gradual improvement in oxygenation and lung compliance is observed. Weaning from mechanical ventilation begins. Day 18: Mr. Smith is successfully extubated, breathing on room air with adequate oxygenation. Day 20: Cardiac medications are adjusted to maintain a stable heart rhythm. Continued physiotherapy for lung rehabilitation. Day 25: Follow-up echocardiogram confirms sustained control of atrial flutter. Mr. Smith is transferred to a step-down unit. Day 30: Mr. Smith was discharged with improved respiratory status and stable cardiac rhythm. Follow-up appointments are scheduled for cardiac and respiratory evaluation.

## 5. Diagnostic assessment

### 5.1. Diagnostic methods

- Physical examination: Irregular heart rhythms, tachypnea, bilateral inspiratory crackles, and peripheral cyanosis were identified during the physical examination.
- Laboratory testing: Arterial blood gas analysis confirmed severe hypoxemia.
- Imaging: Chest X-ray revealed bilateral pulmonary infiltrates consistent with acute respiratory distress syndrome (ARDS). Echocardiogram confirmed atrial flutter.
- Electrocardiogram: Helped diagnose the irregular heart rhythm characteristic of atrial flutter.

### 5.2. Diagnostic challenges

- Financial challenges: Availability of comprehensive testing, especially advanced imaging and specialized consultations.
- Language and cultural barriers: Effective communication with the patient and understanding of medical history might be hindered due to language and cultural differences.

### 5.3. Diagnostic reasoning

- Other potential diagnoses included pneumonia, congestive heart failure, and pulmonary embolism. However, the combination of dyspnea, tachypnea, hypoxemia, and irregular heart rhythms pointed towards atrial flutter complicating ARDS.

### 5.4. Prognostic characteristics

- Staging in this context might not apply to oncology cases. However, ongoing assessment of lung function, cardiac rhythm stability, and overall clinical improvement played a prognostic role in guiding the treatment course and predicting the patient eventual outcome.

## 6. Therapeutic intervention

### 6.1. Types of interventions

#### 6.1.1. Pharmacologic interventions.

- Antiarrhythmic medication (e.g., amiodarone) was administered to control atrial flutter.
- Antibiotics were prescribed empirically to address potential infectious causes of respiratory distress.
- Analgesics and sedatives were used to manage discomfort and anxiety during mechanical ventilation.

#### 6.1.2. Mechanical ventilation.

- It was initiated to support oxygenation and ventilation due to severe respiratory distress and hypoxemia.

#### 6.1.3. Lung rehabilitation.

- Physiotherapy and respiratory exercises were incorporated to improve lung function and promote early weaning from mechanical ventilation.

#### 6.1.4. Administration of intervention.

- Antiarrhythmic medication: Amiodarone was administered intravenously as a loading dose, followed by an oral maintenance dose.
- Antibiotics: Empirical broad-spectrum antibiotics were administered intravenously as per hospital protocol.
- Mechanical ventilation: Positive end-expiratory pressure (PEEP) levels were adjusted based on frequent oxygenation and lung compliance monitoring.

#### 6.1.5. Changes in intervention.

- Antiarrhythmic medication: Dosage adjustments were made to achieve optimal control of atrial flutter while monitoring for potential adverse effects.
- Mechanical ventilation: PEEP levels were modified based on the patient response and evolving lung compliance to improve oxygenation and ventilation.

#### 6.1.6. Rationale for changes.

- The antiarrhythmic medication dosage adjustments were made to maintain stable cardiac rhythm while minimizing the risk of arrhythmia.
- PEEP levels were altered based on the patient evolving lung condition and response to mechanical ventilation to optimize oxygenation and prevent lung injury.

#### 6.1.7. Self-care.

- Mr. Smith was educated about medication adherence, post-discharge follow-up appointments, and lifestyle modifications to manage hypertension and prevent future cardiovascular events.

## 7. Follow-up and outcomes

### 7.1. Clinician-assessed outcomes

- Cardiac rhythm control: Amiodarone administration successfully controlled atrial flutter, as evidenced by restoring normal heart rhythm.
- Respiratory improvement: Mechanical ventilation and appropriate adjustments led to gradual oxygenation and lung compliance improvement.

### 7.2. Patient-assessed outcomes

- Subjective improvement: Mr. Smith reported reduced dyspnea, improved breathing, and enhanced comfort as his respiratory distress lessened.

### 7.3. Follow-up test results

- Echocardiogram: Follow-up echocardiogram confirmed sustained control of atrial flutter and provided insights into cardiac function.
- Arterial blood gas analysis: Repeated analyses indicated improved oxygenation levels.

### 7.4. Intervention adherence and tolerability

- Adherence: Mr. Smith demonstrated an excellent commitment to medication dosing and ventilator support.
- Tolerability: He tolerated antiarrhythmic medication without significant adverse effects, and his response to mechanical ventilation was appropriate.

### 7.5. Adverse and unanticipated events

- Proarrhythmia monitoring: Close monitoring for proarrhythmia, a potential adverse effect of antiarrhythmic medications, was conducted, but no such events were observed.
- Ventilator-associated events: While on mechanical ventilation, measures were taken to prevent ventilator-associated events such as ventilator-associated pneumonia and barotrauma, and no such events occurred.

## 8. Discussion

### 8.1. Strengths in management

- Multidisciplinary approach: Collaboration between cardiology and critical care teams facilitated effective atrial flutter and ARDS management.
- Tailored therapies: Individualized treatment plans considering cardiac and respiratory complexities contributed to positive outcomes.
- Timely interventions: Prompt initiation of antiarrhythmic medication and mechanical ventilation minimized complications.

### 8.2. Limitations in management

- Diagnostic delays: Early recognition of the complex interplay between atrial flutter and ARDS could have led to more efficient management.
- Language barriers: Potential language barriers might have impacted patient communication and comprehension of interventions.

### 8.3. Relevant medical literature

The case report builds upon existing literature by highlighting atrial flutter and ARDS rare coexistence. Similar cases are sparsely documented, emphasizing the need for increased awareness among healthcare practitioners.

### 8.4. The rationale for conclusions

- Atrial flutter complication: The occurrence of atrial flutter complicating ARDS was supported by clinical findings, diagnostic tests, and response to antiarrhythmic medication.
- Cardiopulmonary interaction: The interaction between cardiac arrhythmias and respiratory distress was evident, underscoring the necessity for coordinated management.

### 8.5. Main take-away lessons

• Vigilance in complex cases: This case emphasizes the importance of remaining vigilant for rare complications in critical care scenarios.
• Multidisciplinary collaboration: Effective collaboration between different specialties is crucial for optimal management of complex conditions.
• Tailored interventions: Individualized treatment plans, considering the interplay of multiple factors, can lead to positive outcomes in intricate cases.

## 9. Patient perspective

During my time in the hospital, I felt overwhelmed by the sudden onset of severe breathing difficulties and the irregular heartbeats I was experiencing. The medical team quickly jumped into action, explaining the necessity of mechanical ventilation and antiarrhythmic medication to control my heart rhythm. While the machines and tubes were initially daunting, the nurses’ and doctors’ care and attention reassured me. As the days passed, I noticed improved breathing, and the heart rhythm was gradually controlled. The medical staff kept me informed at every step, patiently answering my questions and involving me in decisions about my care. I’m grateful for their expertise and the individualized approach that helped me regain my health. This experience has taught me the importance of timely intervention and the significance of a supportive healthcare team during times of uncertainty.

## 10. Informed concept

Mr. Smith provided informed consent for the medical interventions and treatments he received during his hospitalization. The medical team thoroughly explained the nature of his condition, the proposed treatments, potential risks and benefits, and alternative options available. They addressed his questions and ensured he had a clear understanding of the procedures and interventions being undertaken. Mr. Smith expressed his understanding of the information provided and willingness to proceed with the recommended course of action. His medical records documented his consent, adhering to ethical and legal patient care and decision-making standards.

## Author contributions

**Conceptualization:** Chukwuka Elendu, Dependable C. Amaechi.

**Data curation:** Chukwuka Elendu, Dependable C. Amaechi.

**Formal analysis:** Chukwuka Elendu, Dependable C. Amaechi.

**Funding acquisition:** Chukwuka Elendu, Dependable C. Amaechi.

**Investigation:** Chukwuka Elendu, Dependable C. Amaechi.

**Methodology:** Chukwuka Elendu, Dependable C. Amaechi.

**Project administration:** Chukwuka Elendu, Dependable C. Amaechi.

**Resources:** Chukwuka Elendu, Dependable C. Amaechi.

**Software:** Chukwuka Elendu, Dependable C. Amaechi.

**Supervision:** Chukwuka Elendu, Dependable C. Amaechi.

**Validation:** Chukwuka Elendu, Dependable C. Amaechi.

**Visualization:** Chukwuka Elendu, Dependable C. Amaechi

**Writing – original draft:** Chukwuka Elendu, Dependable C. Amaechi.

**Writing – review & editing:** Chukwuka Elendu, Dependable C. Amaechi.
